# piRNA in Machine-Learning-Based Diagnostics of Colorectal Cancer

**DOI:** 10.3390/molecules29184311

**Published:** 2024-09-11

**Authors:** Sienna Li, Valentina L. Kouznetsova, Santosh Kesari, Igor F. Tsigelny

**Affiliations:** 1 CureScience Institute, San Diego, CA 92121, USA; applerx@gmail.com (S.L.); vkouznetsova@ucsd.edu (V.L.K.); 2 San Diego Supercomputer Center, University of California San Diego, La Jolla, CA 92093, USA; 3 Pacific Neuroscience Institute, Santa Monica, CA 90404, USA; santosh.kesari@providence.org; 4 Department of Neurosciences, University of California San Diego, La Jolla, CA 92093, USA

**Keywords:** piRNA, machine learning, colorectal cancer, diagnostics

## Abstract

Objective biomarkers are crucial for early diagnosis to promote treatment and raise survival rates for diseases. With the smallest non-coding RNAs—piwi-RNAs (piRNAs)—and their transcripts, we sought to identify if these piRNAs could be used as biomarkers for colorectal cancer (CRC). Using previously published data from serum samples of patients with CRC, 13 differently expressed piRNAs were selected as potential biomarkers. With this data, we developed a machine learning (ML) algorithm and created 1020 different piRNA sequence descriptors. With the Naïve Bayes Multinomial classifier, we were able to isolate the 27 most influential sequence descriptors and achieve an accuracy of 96.4%. To test the validity of our model, we used data from piRBase with known associations with CRC that we did not use to train the ML model. We were able to achieve an accuracy of 85.7% with these new independent data. To further validate our model, we also tested data from unrelated diseases, including piRNAs with a correlation to breast cancer and no proven correlation to CRC. The model scored 44.4% on these piRNAs, showing that it can identify a difference between biomarkers of CRC and biomarkers of other diseases. The final results show that our model is an effective tool for diagnosing colorectal cancer. We believe that in the future, this model will prove useful for colorectal cancer and other diseases diagnostics.

## 1. Introduction

Piwi-interacting RNAs, also known as piRNAs, are RNAs with 24–31 nucleotides found in the germline of many species. They are the largest class of non-coding RNAs (functional RNAs that are not translated into a protein). Studies have shown the role of piRNAs as biomarkers and therapeutic targets for cancer patients [1]. An example of this is piR-36712, whose concentration is negatively correlated with tumor sizes within breast cancer [2]. The functions of piRNAs are still not entirely understood, and they are actively studied as biomarkers in neurodegenerative disease and cancer, including colorectal cancer (CRC) [1].

CRC is currently the third most common cancer diagnosis between men and women, making it a vital area of study [3]. Studies show that CRC is much easier to treat when it is detected early, with more than a 90% 5-year survival rate at a localized stage compared to less than 10% if it has spread to distant parts of the body [3]. Thus, within the field of CRC, detecting biomarkers has been vital to the advancement of treatment within patients. In this paper, we will explore predicting CRC with these biomarkers through machine learning (ML) techniques to aid with the diagnostics of future patients.

Several studies have been conducted in the field of piRNAs and CRC. Qu and colleagues compared piRNAs in healthy individuals, patients with CRC stages I and II, and patients with CRC stages III and IV [4]. Using serum samples from the patients, a reverse transcription quantitative real-time PCR was used to create biomarker panels. These panels were then compared and five differently expressed piRNAs were elucidated as potential biomarkers [4]. Weng and colleagues conducted a similar study using small RNA sequencing [5]. They investigated several piRNAs as possible prognostic biomarkers [5].

Using such research, validated piRNA biomarkers have been suggested. With these data, we have created descriptors for ML models to predict other probable piRNA relations with colorectal cancer. Due to the limited data availability in the piRNA field, we decided to use sequences and their descriptors to predict associations.

piRNAs were initially assumed to be only involved with the reproductive system. However, it was recently realized that various piRNAs tend to have abnormal expressions in other tissues. Recently it was discovered that piRNA can exist not only in the germline, but in other cancer tissues and body fluids [6]. piRNAs have been proven to correlate with tumor cell invasion into distant parts of the body (metastasis). Upregulations of piRNA-823, for example, are associated with distant metastasis in gastrointestinal cancers, including CRC [7].

In other cancers such as breast cancer, piRNAs have been found within tumor cells, indicating association. Although their exact role is not known, because of their irregular expression, piRNAs are thought to have regulating abilities for cancer development and progression [8].

Li and colleagues elucidated irregular piRNA levels in lung-cancer patients as well and found a correlation between piRNA-651 and tumor growth. Using 78 separate lung-cancer patients, they used quantitative real-time PCR to detect the levels of piRNA-651 in tumor cells [9].

Furthermore, Cheng and colleagues concluded that piRNA-651 could be involved with the development of gastric cancer itself. They observed an upregulation of piRNA-651 in cancerous tissues compared to that in noncancerous tissues. The upregulation of piRNA-651 was, in fact, found to be correlated with all gastric, lung, mesothelium, breast, liver, and cervical cancer cell lines. The authors also wrote that multiple piRNAs were found upregulated in these cells, suggesting a significant correlation between piRNA and cancer cells [10].

Liu and colleagues concluded that the dysregulation of piRNA was associated with several diseases, especially cancer tumors and reproductive system diseases [1]. All of these authors discussed that more needs to be known to find the direct correlation and effect that piRNAs have on cancer; however, there is an obvious pattern between piRNAs in cancerous or reproductive diseases. This could be both a dysregulation and an upregulation of a specific piRNA, suggesting that piRNAs could be directly related to tumor development. piRNAs are directly involved in cancer development. In neuroblastoma, piRNA-39980 targets the *JAK3* gene, causing cell proliferation and increasing metastasis [11]. Alternatively, some piRNAs serve as anticancer molecules. For example, piRNA DQ594040 targets the *TNFSF4* gene and inhibits bladder cancer cell proliferation [11].

Several research papers have been published with ML-based diagnostics using small non-coding RNAs as biomarkers. Kang and colleagues, for example, used ML methods for miRNA–disease associations for three types of cancer. They developed a set of descriptors, which were used for disease classification [12]. Xu and colleagues used target genes and pathways to create ML models for Alzheimer’s disease diagnostics [13]. The use of machine learning to explore biomarkers for diseases through small non-coding RNAs has grown in popularity in recent years. In this study, we used similar strategies for CRC diagnostics through piRNAs.

## 2. Results

The results of different model classification algorithms were evaluated based on the confusion matrices. The True Positive Rate (TPR), the False Positive Rate (FPR), the precision, the recall, the F-Measure, the Matthews correlation coefficient (MCC), the area under the receiver-operating characteristic (ROC) curve (AUC), and the area under the precision–recall curve (AUPRC) were all considered (Table 1). These statistical characteristics are derived from the confusion matrix.

### 2.1. Performance Comparison for Different Classifiers through Cross-Validation

**Figure 1:**
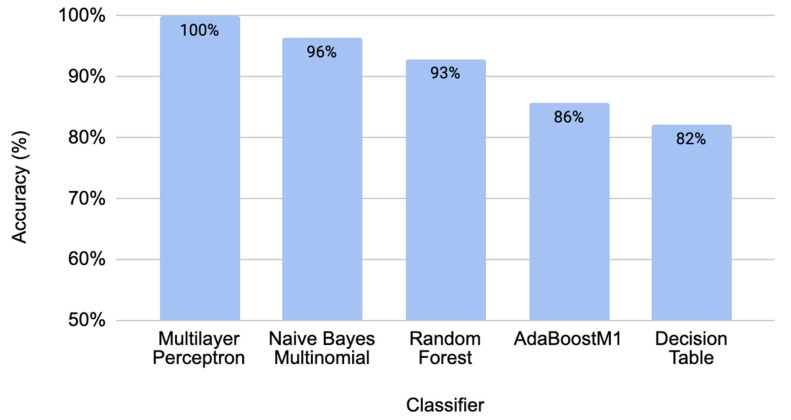
Accuracies of ML model derived through cross-validation for several classifiers.

**Figure 2:**
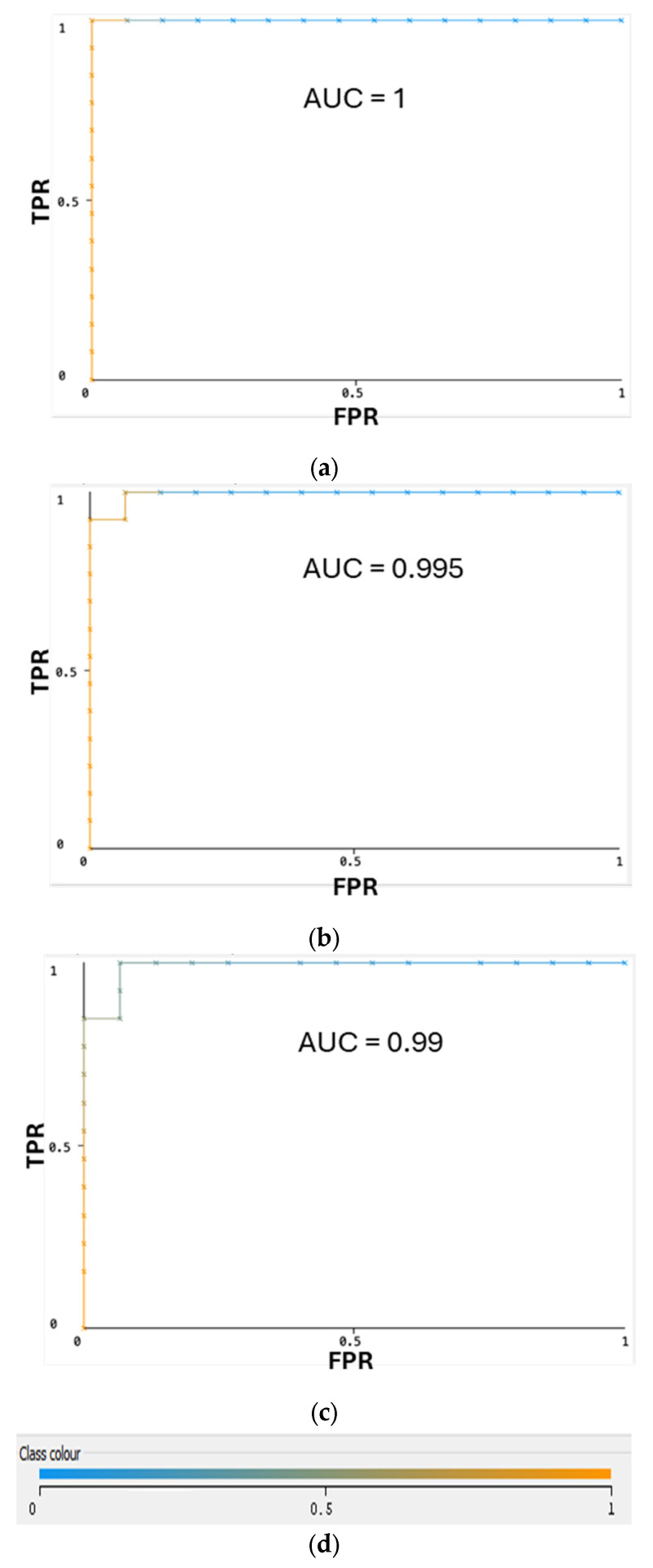
ROC curves for ML classifiers for the testing data set: (**a**) Multilayer Perception; (**b**) Naïve Bayes Multinomial; (**c**) Random Forest. (**d**) Color interpretation of ROC curves. Color represents threshold value set to get the best pair of true FPR/TPR point.

**Figure 3:**
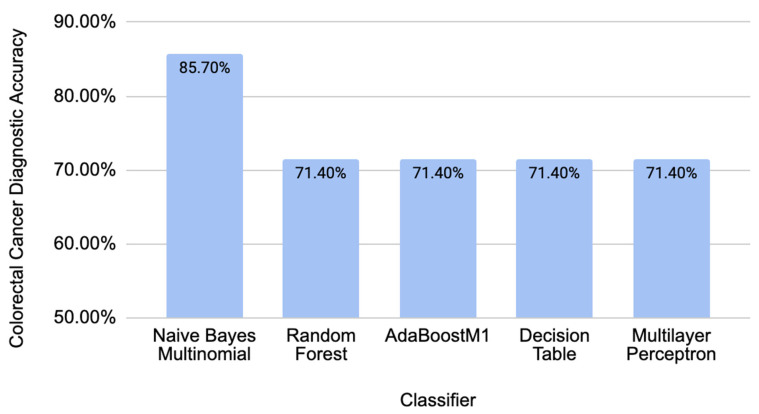
Diagnostic accuracies that were obtained from independent CRC-related data.

Accuracies of 10-fold cross-validation for several best classifiers with the developed ML model are shown in Figure 1. The best-performing models reached over 90% accuracy with the 10-fold cross-validation—ML algorithms such as the multilayer perceptron (MLP)—100%, Naïve Bayes Multinomial—96%, and Random Forest—93%, gave perfect values for all derivatives of the confusion matrix (Table 1). Accuracies for the best classifiers are illustrated in Figure 1. The ROC curves (Figure 2) demonstrate a very high performance of classifiers on the classification thresholds. Overall, the MLP shows the best results in cross-validation, but the Naïve Bayes Multinomial gives the most accurate results in the independent data testing (Figure 3). The entire dataset including all selected and random descriptors was used for cross-validation and resulted in Figure 1 and Figure 2.

### 2.2. Comparison of Different Classifiers’ Performance on Independent CRC-Related Data

To test the validity of our model on an independent new dataset, we used data from piRBase with known associations with CRC. All the piRNAs in this dataset were not present in the piRNAs used for training a model [14]. Taking seven new piRNAs with correspondence to CRC (piR-000335, piR-005132, piR-015481, piR-021520, piR-015551, piR-020980, and piR-002587), we calculated all the corresponding sequence descriptors and tested them in the trained model. The resulting accuracies were calculated by taking the total number of predicted biomarkers over all the piRNAs tested in the independent dataset. Figure 3 summarizes the diagnostic accuracy for the new independent data of the best-performing classifiers used from previous data to train the model. These accuracies show that our model can make diagnostics with independent data related to CRC.

### 2.3. Comparison of Different Classifiers’ Performance on Independent CRC-Unrelated Data

**Figure 4:**
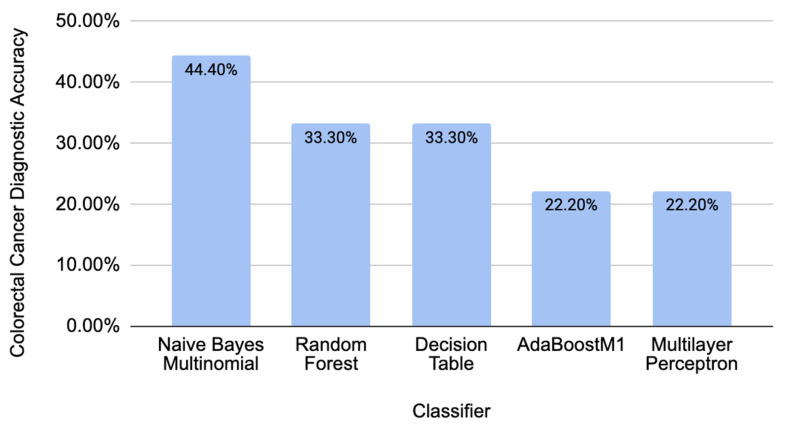
Diagnostic accuracies that were obtained using CRC-unrelated data.

To further test the validity of our model, we ran data from piRBase with known associations to breast cancer to analyze the data with no connection to CRC [14]. Taking nine piRNAs (piR-932, piR-31106, piR-34377, piR-34736, piR-35407, piR-36026, piR-36249, piR-36318, and piR-36743), we once again calculated all the corresponding sequence descriptors to test them in the trained model (Figure 4). Overall, these obtained accuracies are much lower than the accuracies of the classifiers with the initial CRC-related data, showing that the model can detect the difference between data that have a correlation (Figure 3) and data that are not correlated to CRC (Figure 4).

## 3. Discussion

Our data were gathered from a study that isolated potential biomarkers for CRC using quantitative real-time PCR (qRT-PCR). The authors [3] extracted piRNAs from the blood, saliva, mucus, and/or tissue of patients. With this data, we developed the ML model for the diagnostics of CRC. Using our ML model for unknown data with the same set of descriptors as we used in training the piRNA dataset, we can find that the patient could have CRC and suggest further testing.

CRC remains to be one of the world’s deadliest diseases. To date, the distant stage of CRC only has less than a 10% 5-year survival rate [3]. CRC is usually found through a colonoscopy procedure where the rectum and entire colon are observed under a colonoscope [3,15]. However, many post-procedure complications may arise due to the nature of the procedure, and for many at-risk groups such as pregnant women, people with pre-existing diseases, or the elderly, colonoscopies can cause dehydration or electrolyte problems [16]. CRC research also shows that patients younger than 50 who are diagnosed with CRC tend to have a more advanced stage of the disease [17]. Colonoscopies are traditionally conducted after the age of 50, making the screening and risk assessment in younger patients less common and harder to find earlier. Thus, finding reliable biomarkers for CRC is crucial.

piRNAs, which are found in somatic cells, can maintain germline DNA integrity, silence transcription, and suppress the translation of cancer-related genes [18]. As such, piRNA can be used as a biomarker of cancers including CRC.

This paper shows the importance of biological data for the early detection of CRC and as an early diagnosis biomarker. Observing the piRNAs found with a correlation with CRC, we first created a multinomial model that achieved over 96% accuracy of CRC elucidation. The sequence descriptors we generated were determined through initial selection, and our models could become a basis for future research into the field of piRNAs for multiple diseases. This model was then tested using independent testing data and achieved over an 85% accuracy of CRC elucidation with the independent CRC data and under 50% for non-CRC data related to breast cancer.

It is important to address the limitations of this study. For example, the lack of information currently available on piRNAs, such as gene targets, can indicate that this model can be improved in the future. However, with the patterns detected from the piRNA sequence descriptors, we can conclude that ML is an effective method for the use of piRNAs as biomarkers for diagnostics.

## 4. Materials and Methods

### 4.1. Classification Model

**Figure 5:**
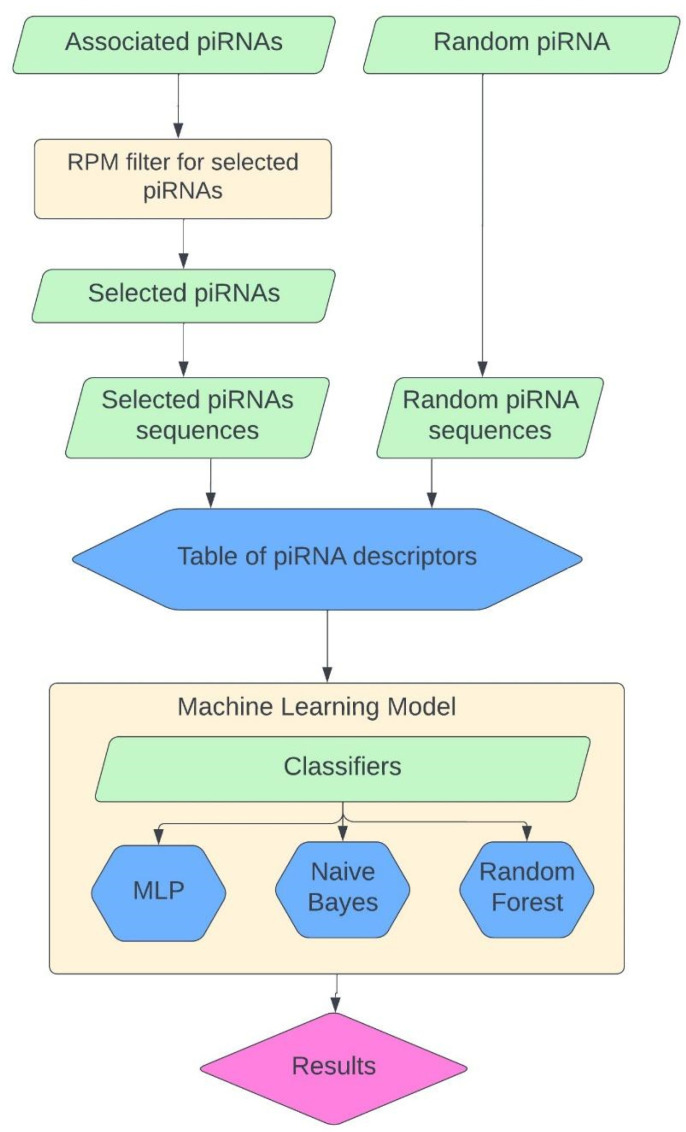
Flowchart of the Method. The list of piRNAs associated with a disease was collected from experimental studies, then it was filtered by the values of fold changes of RPM in relation to normal subjects. Also, the equivalent number of random piRNAs was selected. The sequences were extracted from both datasets and used for the creation of sequence descriptors. These descriptors were used for the development of an ML model using various classifiers. The ML model with the best accuracy was used for further exploration of new data.

Utilizing known associations with piRNA and CRC, we developed a classification model (Figure 5) using Waikato Environment for Knowledge Analysis (WEKA) software [19]. We selected 13 different piRNAs that have shown a correlation to CRC [4] (piR-001312, piR-004150, piR-004153, piR-009295, piR-014620, piR-016677, piR-017716, piR-017723, piR-017724, piR-020326, piR-020365, piR-020388, and piR-020829), and then we extracted 13 random piRNAs from the piRNA database piRNAdb [14]. The ML model was then created using sequence descriptors like the set used in another study [12]. Additional descriptors were added for motifs in the first and last 5 nucleotides of piRNAs because it is known that these molecules have differences in starting and ending sequences. Figure 5 displays the preparation of the piRNA descriptor table with associated piRNA sequences, filtered by piRNA reads per million (RPM), fold change (FC) values, and random piRNA sequences. This table is then associated with several classifiers to build an ML model to predict if piRNAs can be used for the diagnostics of CRC. We compared these classifiers to find the most accurate models (Figure 5).

### 4.2. Sequence Descriptor System

We analyzed “reads per million (RPMs) clean tags” of piRNAs in control individuals and CRC patients and their fold changes (FC) values. We extracted ratios of RPMs from 2 different groups, group A—CRC patients in stages I and II, and B—CRC patients in stages III and IV, with a combined total of 220 CRC patients [4]. When we analyzed the RPM and FC values of the expressions of piRNA in CRC patients related to healthy individuals, we found that the FC values are surprisingly very close for all piRNAs in the A and B groups. Eventually, we used the list of 13 piRNAs, having significant FC in both groups.

Alongside selected CRC-related piRNAs, 13 non-associated piRNAs were randomly selected from piRNAdb [14]. We selected these piRNAs using a random number generator without repetitions. The 13 associated values were labeled “selected”, while the non-associated values were labeled “random”. A set of descriptors was calculated from the piRNA sequences found in piRNAdb [14]. We used a table of sequence descriptors applied in similar studies with small non-coding RNAs for cancer classification [12].

We developed a Python script to evaluate all input sequences, calculate the numerical values related to nucleotides motifs, symmetry, and repetitions, and used them as sequence descriptors, which included: the number of all nucleotides in piRNA, the numbers of each separate nucleotide (A, U, C, and G in this case), the frequency of each nucleotide, the mean mass of each nucleotide, the number of hydrogen bonds, and symmetry, which was calculated by comparing the sequence with a reflected version of the sequence and counting the number of nucleotides that were the same. Other values were created to calculate every 2-, 3-, and 4-base pair motifs found in the entire piRNA sequence [12]. The same strategy was used to compare the first 5- and the last 5-base pairs, respectively. Each pattern we searched for was then used as a descriptor for the ML model. In total, 1020 descriptors were created. This system can be replicated in any other study relating to small non-coding RNAs and disease classification.

The Python script is available upon request.

We used the InfoGainAttributeEval function to select the sequence descriptors that contribute the most to disease classification. This greatly reduced the 1020 descriptors to 27 that made the most contributions to building the model (Table 2). The fragment of the large table of descriptors is presented in Table 3.

### 4.3. Classifier Descriptions

The types of ML classifiers we used include the Multilayer Perceptron, Naïve Bayes, Decision Table, Logistic Regression, K-Nearest Neighbor, Artificial Neural Networks/Deep Learning, and Support Vector Machine.

The Multilayer Perceptron is a feed-forward artificial neural network. There is an input layer, an output layer, and an arbitrary number of hidden layers in between. Prediction and classification are completed by the output layer.

The Naïve Bayes Multinomial calculates the probability for each option and creates a prediction of the output with the highest probability. The probabilities culminate through each predictor.

Random Forest uses multiple decision trees to reach a single result. The output is the class that the majority of the decision trees reach.

AdaBoostM1, also known as Adaptive Boosting, uses multiple weak or base learners to classify the data. For example, this includes many single-layer decision trees.

The Decision Table documents all possible actions and outcomes to reach one outcome.

## 5. Conclusions

We propose a descriptor system using the piRNA parameters and sequence descriptors to develop ML models for colorectal cancer. We want to note that the current article is the first publication describing the use of piRNA and ML for the diagnostics of CRC.

We compared several classifiers such as the Random Forest, Naïve Bayes Multinomial, AdaBoostM1, Multilayer Perceptron, and Decision Table. Each ML model was able to respond with more than 90% accuracy, and most models were able to correctly classify independent data with more than 70% accuracy. Furthermore, data not related to CRC achieved much lower accuracies, showing that our model can be highly selective in CRC elucidation. With this proof, we created an ML model that can explore the piRNA correlation with CRC. The results show that our model can be an effective tool for diagnosing colorectal cancer. The current model trained on the limited number of piRNAs is proof of the principle. We would not recommend using it in clinical practice right away. Currently, we are working on models that can be trained on significantly more piRNAs related to CRC and other cancers. These would be useful in a clinical environment.

## Figures and Tables

**Table 1:** Performance comparison for multiple different classifiers on the dataset.

Classifier	TPR	FPR	Precision	Recall	F-Measure	MCC	AUC	AUPRC
Multilayer Perceptron	100%	0%	100%	100%	100%	100%	100%	100%
Naïve Bayes Multinomial	96.40%	3.10%	96.70%	96.40%	96.40%	93.10%	99.50%	99.50%
Random Forest	92.90%	8.20%	93.70%	92.90%	92.80%	86.40%	99.00%	99.10%
AdaBoostM1	85.70%	15.50%	86.30%	85.70%	85.60%	71.70%	89.20%	90.30%
Decision Table	82.10%	19.60%	83.50%	82.10%	81.80%	65.10%	71.50%	71.40%

**Table 2:** Sequence descriptors that have the most informational impact on the ML model.

Descriptor	Explanation
C	Number of C nucleotides
C/N	Frequency of C nucleotides
CU	Number of CU dinucleotides
UUC	Number of UUC trinucleotides
CGC	Number of CGC trinucleotides
5sCAG	Number of CAG trinucleotides in the first 5 nucleotides of piRNA
5sAAG	Number of AAG trinucleotides in the first 5 nucleotides of piRNA
5sGGU	Number of GGU trinucleotides in the first 5 nucleotides of piRNA
5sGGC	Number of GGC trinucleotides in the first 5 nucleotides of piRNA
5eCA	Number of CA dinucleotides in the last 5 nucleotides of piRNA
5eUGA	Number of UGA trinucleotides in the last 5 nucleotides of piRNA
5eGGA	Number of GGA trinucleotides in the last 5 nucleotides of piRNA
5eAGG	Number of AAG trinucleotides in the last 5 nucleotides of piRNA
AGGC	Number of AGGC four nucleotides’ motifs
AUCA	Number of AUCA four nucleotides’ motifs
GAAA	Number of GAAA four nucleotides’ motifs
GAGU	Number of GAGU four nucleotides’ motifs
GGCA	Number of GGCA four nucleotides’ motifs
GUAG	Number of GUAG four nucleotides’ motifs
GUGU	Number of GUGU four nucleotides’ motifs
CUUC	Number of GUUC four nucleotides’ motifs
UAAA	Number of UAAA four nucleotides’ motifs
UCCA	Number of UCCA four nucleotides’ motifs
UCCC	Number of UCCC four nucleotides’ motifs
UCUG	Number of UCUG four nucleotides’ motifs
UUGU	Number of UUGU four nucleotides’ motifss

**Table 3:** Fragment of sequence descriptors for three selected piRNAs.

piRNA	A	G	C	U	AA	GG	UU	CC	AAA	GGG	UUU	CCC	N	A/N	G/N	C/N	U/N	Mass/N
piR-001312	7	8	3	6	0	4	1	0	0	0	0	0	29	0.24	0.28	0.1	0.21	111.88
piR-004150	7	5	9	2	2	1	0	3	0	0	1	0	30	0.23	0.17	0.3	0.07	98.44
piR-004153	9	5	7	4	1	2	0	1	1	0	0	0	30	0.3	0.17	0.2	0.13	108.45

## Data Availability

Data are contained within the article. A code is available upon request.
